# Genetic predisposition to smoking and the risk of Carpal Tunnel Syndrome: a mendelian randomization study

**DOI:** 10.1016/j.clinsp.2026.100950

**Published:** 2026-04-11

**Authors:** Qi Zeng, Xuepeng Rao, Weiwen Hu, Haichao Chao, Yu Cheng, Guanghao Zheng

**Affiliations:** a The Second Affiliated Hospital, Jiangxi Medical College, Nanchang University, Nanchang, China; b Queen Mary University of London, Jiangxi Medical College, Nanchang University, Nanchang, Jiangxi, China; c Department of Urology, The Second Affiliated Hospital of Nanchang University, Nanchang, China; d Department of Medicine, Jiangxi Medical College, Nanchang University, Nanchang, Jiangxi Province, China; e Nanchang University, Nanchang, Jiangxi Province, China

**Keywords:** Carpal tunnel syndrome, Smoking, Nicotine dependence, Mendelian randomization analysis, Genetic epidemiology, Causality, Risk Factors, Genome-Wide Association Study

## Abstract

**Background:**

Carpal Tunnel Syndrome (CTS) is a common entrapment neuropathy leading to pain and functional impairment. While smoking has been proposed as a modifiable risk factor, evidence from observational studies remains inconsistent due to potential confounding and reverse causality.

**Method:**

The authors performed a two-sample Mendelian Randomization (MR) analysis to assess the causal relationship. Genetic instruments for five smoking phenotypes were derived from large-scale Genome-Wide Association Studies (GWAS). Summary-level data for CTS were obtained from the FinnGen Consortium (n = 480,201). The Inverse Variance Weighted (IVW) method was the primary analysis, supplemented by four other MR methods and a suite of sensitivity analyses to assess robustness and pleiotropy.

**Results:**

Genetically predicted smoking propensity was associated with a higher risk of CTS. Specifically, genetic predisposition to smoking initiation (ORMR-IVW = 1.53; 95% CI 1.33–1.76; p < 0.001) and heavier cigarette consumption (ORMR-IVW = 1.52; 95% CI: 1.31–1.77; p < 0.001) significantly increased CTS risk. The estimate for current smoking was particularly strong (ORMR-IVW=3.96; 95% CI 2.20–7.12; p < 0.001). Sensitivity analyses were largely consistent, though some heterogeneity and potential pleiotropy were detected for specific phenotypes.

**Conclusions:**

This MR study supports a causal role of smoking in the development of CTS. These findings underscore the importance of smoking cessation as a potential preventive measure against CTS and should be integrated into patient counseling and public health strategies.

## Highlights

• Genetics establishes smoking as a cause of Carpal Tunnel Syndrome.
• Genetic predisposition to smoking raises the risk of Carpal Tunnel Syndrome.
• Finding is robust to genetic confounding.
• Quitting smoking is key to preventing Carpal Tunnel Syndrome.

## Introduction

Carpal Tunnel Syndrome (CTS) is one of the most prevalent peripheral nerve entrapment syndromes, primarily caused by the compression of the median nerve as it passes through the narrow carpal tunnel.^1^ Although the precise pathophysiology remains unclear, factors such as edema, tendon inflammation, hormonal changes, and physical activity may exacerbate nerve compression, leading to pain, weakness, and functional impairment.^2^ Epidemiologically, it is estimated that one in ten individuals will experience CTS during their lifetime.^3^ The condition is particularly prevalent among women around the age of 50, with its incidence increasing significantly with age, especially in elderly women.^4^ CTS not only compromises the physical health of affected individuals but also imposes substantial psychological and economic burdens.^5^, ^6^, ^7^ Consequently, a comprehensive understanding of CTS is crucial for the development of effective prevention and treatment strategies.

Smoking continues to represent a major public health challenge.^8^ Global estimates indicate that approximately 1.18 billion individuals smoke, underscoring the need for a thorough investigation into the detrimental health effects of smoking.^9^ Over the past two decades, numerous studies have identified a potential association between smoking and CTS, although the findings have been inconsistent.^10^^,^^11^ A meta-analysis of cross-sectional data suggests that smokers are nearly twice as likely to develop CTS compared to non-smokers.^10^ However, an alternative meta-analysis fails to support this association.^11^ These findings primarily derive from observational studies, which are vulnerable to confounding bias and reverse causality, limiting the ability to draw definitive causal conclusions and highlighting the need for further exploration.

Mendelian Randomization (MR) is a genetic epidemiology method that uses genetic variation as an Instrumental Variable (IV) to investigate causal relationships between different traits.^12^^,^^13^ This method is based on Mendel's laws of inheritance and is similar to a Randomized Controlled Trial (RCT), using genes as a natural experiment to help overcome confounding factors in observational studies.^14^ Here, the authors conducted an MR study to investigate the causal relationship between smoking and CTS.

## Materials and methods

### Study design

**Fig. 1:**
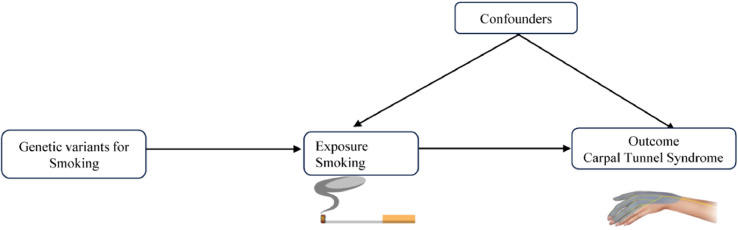
Overview of the Mendelian randomization study design.

The authors utilized two-sample MR to evaluate the association between smoking and CTS. This MR study was conducted in accordance with the STROBE-MR guidelines. The core assumptions of the two-sample MR analysis are based on three fundamental conditions. First, a robust and significant correlation between the IVs, which are SNPs, and smoking. Second, the IVs are uncorrelated with any confounding factors. Third, the exclusivity assumption asserts that the IV influences the outcome exclusively through the exposure factor, with no contribution from alternative pathways. A schematic of the MR study design is presented in Fig. 1. The validity of these assumptions is essential to ensuring that causal effect estimates remain unbiased.

### Data sources

All GWAS data utilized in this study were obtained from European cohorts, with publicly available data from these populations being employed. This study used publicly available summary-level GWAS data. Ethical approval and informed consent were obtained in all original studies. No additional ethical approval was required for this analysis. The CTS GWAS dataset was sourced from the FinnGen consortium (https://r12.finngen.fi/), which includes genetic and health information from 480,201 Finnish participants of European descent. This study examined five smoking-related exposures: “Smoking initiation”, “individual began smoking regularly”, “cigarettes per day (past and current)”, “Current tobacco smoking”, and “Smoking status: Never”. The “Smoking initiation” group includes the age at which individuals first began smoking regularly.^15^ The “individual began smoking regularly” group indicates a binary phenotype, reflecting whether an individual has ever smoked regularly. “Cigarettes per day (past and current)” quantifies the daily number of cigarettes smoked, measuring the intensity of smoking. The “Never smokers” group consists of participants who have never smoked, while the “Current smokers” group includes individuals with a history of smoking who are currently smoking. The datasets used in this study were carefully selected based on their relevance to the research objectives and the availability of comprehensive data. These datasets have demonstrated their capacity to provide high-quality genetic information regarding smoking-related phenotypes and CTS. Detailed information about the data sources is presented in Table 1.

**Table 1:** Detailed information about these datasets.

Phenotype	Sample Size	GWAS ID	Ancestry
Smoking initiation	3383,199	GSCAN	European
Individual began smoking regularly	728,826	GSCAN	European
Cigarettes per day (past and current)	784,353	GSCAN	European
Current tobacco smoking	462,434	ukb-b-223	European
Smoking status: Never	359,706	ukb-d-20,116_0	European
Carpal tunnel syndrome	480,201	finngen_R12_G6_CARPTU	European

### Genetic instrument selection

The selection of IVs is pivotal in the two-sample MR analysis. Initially, smoking-associated SNPs that reached the genome-wide significance threshold (p < 5 × 10^–8^) were screened. An LD coefficient threshold of *r*² < 0.001 within a 10,000 kb window was applied to satisfy the first association assumption. Simultaneously, the authors calculated the statistical strength measure (F-statistic) for the remaining SNPs, where F = R²(N-2) / (1 - R²) and R² = 2 × EAF × MAF × β². In this formula, EAF refers to the effect allele frequency, MAF to the minor allele frequency, β represents the estimated effect size of the effect allele on the exposure, and N denotes the sample size. This statistic reflects the strength of the influence each IV has on the exposure phenotype. SNPs with F < 10 were excluded to avoid bias due to weak instruments. Finally, the selected SNPs were uploaded to the LDtrait tool (https://ldlink.nih.gov/?tab=ldtrait) to control for potential confounding factors.

### Statistical analysis

The “TwoSampleMR (0.6.8)” and “MRPRESSO (1.0)” packages were used to perform MR analysis in R software version 4.4.2, with visualization achieved through the “forestploter (1.1.2)” package. Five different methods were employed, including Inverse Variance Weighting (IVW), MR-Egger, weighted median, simple mode,^16^ and weighted mode,^17^ to assess the causal relationship between smoking and CTS. IVW is a robust method that assumes effective IVs and balanced pleiotropy.^18^ Consequently, IVW was chosen as the primary method of analysis. For features containing a single IV, the Wald ratio test was applied to estimate the association between the IV and each phenotype. For features involving multiple IVs, the standardized IVW estimate was used as the primary method. This method combines the Wald ratio for each SNP with the outcome, yielding a summary causal estimate. MR effect estimates were reported using Odds Ratios (ORs) and 95% Confidence Intervals (95% CIs). Potential heterogeneity across data sources and pleiotropy due to confounding factors may introduce bias in causal effect estimation. To address these concerns, Cochran's *Q* test was used to assess heterogeneity in the IVW and MR-Egger methods, the MR-Egger intercept was used to evaluate pleiotropy,^19^ and the MR-PRESSO method was employed to detect and correct pleiotropy outliers.^20^ The “leave-one-out” method was applied to determine whether any SNP significantly impacted the causal relationship between the exposure and the outcome. Statistical power was calculated using https://shiny.cnsgenomics.com/mRnd/.

## Result

### Results of instrumental variables

The SNPs used for each smoking-related phenotype in the MR analysis are detailed in the Supplementary Table 1. Specifically, the final analysis included 202 SNPs for smoking initiation, 9 for individual began smoking regularly, 40 for cigarettes per day, 26 for current tobacco smoking, and 64 for smoking status: never. The mean F-values were 41.98 for smoking initiation, 42.81 for beginning to smoke regularly, 74.84 for cigarettes per day, 41.19 for current tobacco smoking, and 42.31 for smoking status: never. These high values indicate that the present results are unlikely to be biased by weak instruments.

### Causal relationship between smoking and CTS

**Fig. 2:**
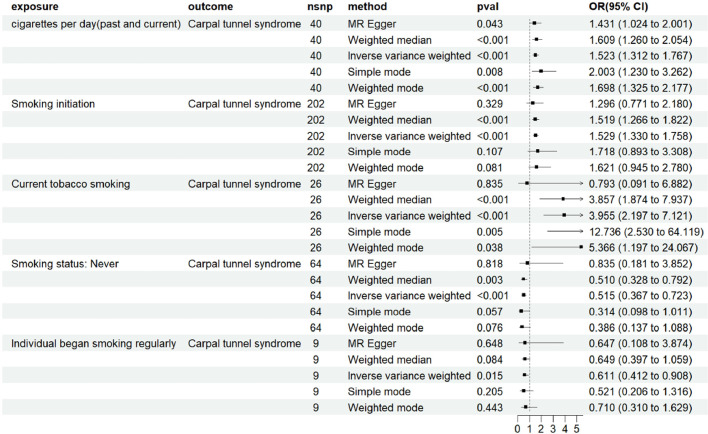
Forest plot for MR estimation of the causal relationship between smoking and CTS. CTS, Carpal Tunnel Syndrome; MR, Mendelian randomization.

**Fig. 3:**
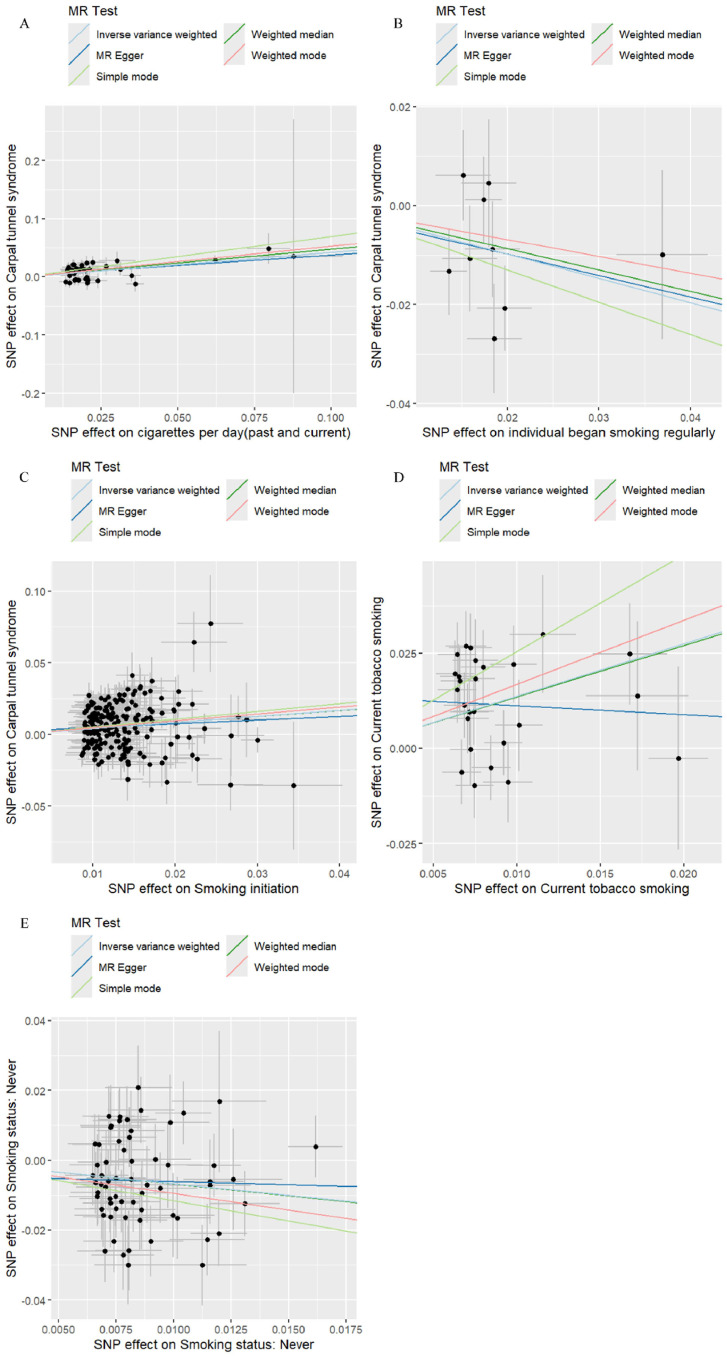
MR scatter plots for the associations of smoking and CTS. (A) Scatter plots for the causal effects of cigarettes per day (past and current) and CTS. (B) Scatter plots for the causal effects of individual began smoking regularly and CTS. (C) Scatter plots for the causal effects of Smoking initiation and CTS. (D) Scatter plots for the causal effects of Current tobacco smoking and CTS. (E) Scatter plots for the causal effects of Smoking status (Never) and CTS. CTS, Carpal Tunnel Syndrome; MR, Mendelian randomization.

Using the IVW method as the primary analysis, the results indicate a positive causal relationship between smoking initiation and increased CTS risk (IVW: OR = 1.529; 95% CI 1.330–1.758, p < 0.001). Heavier cigarette consumption (“cigarettes per day [past and current]”) and current tobacco smoking were also significantly associated with higher risk of CTS (IVW: OR = 1.523; 95% CI 1.312–1.767, p < 0.001; and IVW: OR = 3.955; 95% CI 2.197–7.121, p < 0.001, respectively). Compared to non-smokers, smokers had a 52.3% increased risk of CTS. The Odds Ratios (ORs) derived from MR-Egger, weighted median, simple mode, and weighted mode analyses were broadly consistent with the IVW estimates (Fig. 2). Fig. 3 displays a scatter plot illustrating the consistency and fit of the various analysis methods.

As a sensitivity analysis, genetic predisposition to “Smoking status: Never” was inversely associated with CTS risk (IVW: OR = 0.515, 95% CI 0.367–0.723, p < 0.001). This estimate is largely complementary to that for smoking initiation, providing consistent evidence that smoking increases CTS risk. Conversely, the result for “individual began smoking regularly” (IVW: OR = 0.611; 95% CI 0.412–0.908, p = 0.015) was paradoxical and inconsistent with the overall findings. Given the small number of instruments (n = 9) for this phenotype, this result is likely unreliable and may be driven by weak instrument bias or pleiotropy.

### Assessment of pleiotropy, heterogeneity, and sensitivity

Sensitivity analyses were conducted to assess the robustness of the findings (Table 2). The MR-Egger intercept test did not show significant directional pleiotropy for most traits (all p > 0.05). However, the MR-PRESSO global test indicated significant evidence of horizontal pleiotropy for 'cigarettes per day' (p < 1 × 10^–4^), 'current tobacco smoking' (p = 0.030), and 'smoking status: never' (p = 0.005). Notably, the MR-PRESSO outlier test did not identify any specific SNPs as outliers for these exposures. Cochran's *Q* test revealed significant heterogeneity in the SNP-specific estimates for 'smoking initiation', 'current tobacco smoking', and 'smoking status: never' (all p < 0.05). The results of the leave-one-out analysis confirmed that no single SNP was driving the causal associations (Supplementary Fig. 2). Funnel plots were used to visualize the symmetry of SNP effects (Supplementary Fig. 1).

**Table 2:** Sensitivity analysis of the associations between Carpal tunnel syndrome and smoking.

Exposure	Outcome	Methods	Heterogeneity Statistics			Horizontal Pleiotropy		MR-PRESSO	
			Q	I^2^	p	Egger Intercept	p	Outlier SNPs	P (Global Test)
Cigarettes per day (past and current)	Carpal tunnel syndrome	IVW	43.977	38	0.233			NA	<1e-04
		MR Egger	44.168	39	0.262	0.002	0.687		
Smoking initiation	Carpal tunnel syndrome	IVW	331.911	200	0.000			NA	0.319
		MR Egger	332.607	201	0.000	0.002	0.518		
Current tobacco smoking	Carpal tunnel syndrome	IVW	38.373	24	0.032			NA	0.030
		MR Egger	42.027	25	0.018	0.014	0.144		
Smoking status: Never	Carpal tunnel syndrome	IVW	96.119	62	0.004			NA	0.005
		MR Egger	96.748	63	0.004	−0.004	0.527		
Individual began smoking regularly	Carpal tunnel syndrome	IVW	9.678	7	0.208			NA	0.306
		MR Egger	9.683	8	0.288	−0.001	0.951		

## Discussion

This is a two-sample MR study that provides genetic evidence supporting a causal relationship between lifelong smoking behaviors, particularly current smoking status, and an increased risk of CTS. By using genetic variants as IVs, this approach strengthens causal inference and helps mitigate the confounding and reverse causation biases that have plagued previous observational studies. The robustness of these primary findings across multiple sensitivity analyses enhances their reliability. However, a nuanced interpretation is required, particularly in light of a paradoxical finding and the need to elucidate a specific biological pathway for CTS.

The MR results add a crucial layer of evidence to a field marked by inconsistent observational findings. Several large cross-sectional and cohort studies have reported a significant association between smoking and CTS, even after adjusting for BMI and socioeconomic status.^21^^,^^22^ Conversely, other case-control and retrospective studies, particularly after extensive covariate adjustment or in non-European populations, found no significant relationship.^23^, ^24^, ^25^, ^26^, ^27^ The inherent limitations of these observational designs ‒ such as residual confounding, self-reporting bias in smoking status, and the case-control design's susceptibility to recall bias ‒ likely account for these discrepancies.

While observational studies have suggested a link between smoking and CTS, establishing a direct causal pathway requires a nuanced discussion of biological plausibility that is specifically tailored to the unique pathophysiology of CTS. The authors posit that smoking may act not as a sole cause, but as a critical effect modifier that significantly lowers the median nerve's threshold for compression.^28^ The carpal tunnel is a rigid, non-compliant osteofibrous canal.^2^ Chronic smoking-induced endoneurial hypoxia and systemic subclinical inflammation could lead to subtle, persistent edema of the flexor tendon synovium.^29^^,^^30^ Within this confined space, even a minor increase in synovial volume can disproportionately elevate pressure on the median nerve, initiating a cascade of localized microvascular compromise and demyelination.^31^^,^^32^ This model positions smoking as a potent systemic insult that exacerbates the consequences of other local mechanical risk factors, providing a more specific pathway to CTS.

The magnitude of the causal estimate for current smoking (OR∼4.0) is substantial and necessitates a critical appraisal. While the proposed synergistic model provides a biological framework for a strong effect, an odds ratio of this scale ‒ surpassing that of many established risk factors ‒ also raises the possibility of methodological influences. The significant heterogeneity and signals of horizontal pleiotropy indicate that the genetic instruments may not be perfectly specific. It is plausible that these variants also influence a constellation of correlated social, behavioral, and occupational traits (e.g., manual labor, socioeconomic status), which are themselves independent risk factors for CTS. This residual pleiotropy could conflate the direct effect of smoking with these ancillary pathways, thereby inflating the observed estimate. Therefore, the most prudent interpretation is that the reported OR likely represents an upper bound of the true causal effect, encapsulating both a genuine biological relationship and a component of genetic confounding.

While the present study provides evidence supporting a causal relationship, several limitations must be considered when interpreting the results. First, the threat of residual horizontal pleiotropy remains a principal concern. Although the authors employed robust sensitivity analyses, the significant global test results from MR-PRESSO and the observed heterogeneity for key exposures indicate that the genetic instruments may influence CTS through pathways not entirely mediated by smoking behaviors. This potential pleiotropy could bias the causal estimates, particularly inflating the magnitude of the remarkably large odds ratio observed for current smoking. Second, the authors identify as a serious limitation the paradoxical and statistically significant protective association observed for the “individual began smoking regularly” phenotype (OR = 0.61), which directly contradicts the core hypothesis and established biological knowledge. The authors attribute this likely false signal primarily to weak instrument bias, as this analysis relied on a limited set of only 9 SNPs and a complex definition of this trait in the source GWAS, making it highly susceptible to bias. Given the severity of this limitation, the authors explicitly state that this result should be disregarded as counterevidence, and the authors emphasize that it underscores the critical importance of using powerful, well-defined genetic instruments in MR analyses. Third, the generalizability of these findings is constrained by the exclusive use of GWAS data from individuals of European ancestry. The genetic architecture of both smoking behaviors and CTS may differ across populations, necessitating validation in diverse ethnic groups. Fourth, the authors were unable to investigate potential sex-specific effects due to the lack of publicly available sex-stratified GWAS summary statistics. This is a notable gap, given the established differences in CTS prevalence between males and females, which may reflect distinct underlying risk pathways. Finally, sample overlap between the exposure GWAS and the FinnGen outcome cohort, while a common issue in two-sample MR, may introduce bias and inflate type I error rates. Although the mean F-statistics for the instruments were above conventional thresholds, suggesting minimal weak instrument bias on average, the potential impact of unquantifiable sample overlap persists.

## Conclusion

In conclusion, this study utilized a two-sample MR analysis, which identified a causal relationship between smoking and CTS. Specifically, smoking may contribute to both the incidence and progression of CTS. Consequently, the influence of tobacco use should be integrated into strategies for the prevention and management of CTS. Additionally, further investigation is warranted to elucidate the biological mechanisms driving this association.

## Data availability

All data used in this study are publicly available. Summary statistics were obtained from publicly accessible datasets, including the IEU OpenGWAS database and the FinnGen consortium.

## Ethics approval and consent to participate

This study falls under the exemption criteria specified in Section 4 of the People’s Republic of China’s “Notice on the Implementation of Ethical Review Measures for Life Science and Medical Research”. It exclusively utilized publicly available, anonymized data from GWAS, which does not involve sensitive personal information or pose harm to individuals. As the research does not involve interventions, human biological samples, or activities related to genetic manipulation or reproductive cloning, and all data used were in compliance with applicable laws and terms of use, ethical approval was not required.

## Clinical trial number

Not applicable.

## Authors’ contributions

Study conception and design: Guanghao Zheng; Data analyses: Xuepeng Rao and Qi Zeng; Draft preparation: Guanghao Zheng, Qi Zeng, Yu Cheng and Xuepeng Rao; Statistical analysis: Haichao Chao; Supervision of the study: Guanghao Zheng. All authors reviewed the manuscript.

## Funding

This study was funded by the Jiangxi Provincial Health Commission project (2018A385, 202310034), Jiangxi Provincial academic and technical leaders training program (20225BCJ22009), and the 10.13039/501100001809National Natural Science Foundation of China (82260598).

## Conflicts of interest

The authors declare no conflicts of interest.

## Appendix Supplementary materials

Image, application 1

Image, application 2

Image, application 3

## References

[1] England J.D. (1999). Entrapment neuropathies. Curr Opin Neurol.

[2] Padua L., Coraci D., Erra C., Pazzaglia C., Paolasso I., Loreti C. (2016). Carpal tunnel syndrome: clinical features, diagnosis, and management. Lancet Neurol.

[3] Practice parameter for carpal tunnel syndrome (summary statement) (1993). Report of the quality standards subcommittee of the American academy of neurology. Neurology.

[4] Atroshi I., Gummesson C., Johnsson R., Ornstein E., Ranstam J., Rosén I. (1999). Prevalence of carpal tunnel syndrome in a general population. JAMA.

[5] Atroshi I., Gummesson C., Johnsson R., Sprinchorn A. (1999). Symptoms, disability, and quality of life in patients with carpal tunnel syndrome. J Hand Surg Am.

[6] Wilson d'Almeida K., Godard C., Leclerc A., Lahon G. (2008). Sickness absence for upper limb disorders in a French company. Occup Med.

[7] Foley M., Silverstein B., Polissar N. (2007). The economic burden of carpal tunnel syndrome: long-term earnings of CTS claimants in Washington State. Am J Ind Med.

[8] Dai X., Gil G.F., Reitsma M.B., Ahmad N.S., Anderson J.A., Bisignano C. (2022). Health effects associated with smoking: a Burden of Proof study. Nat Med.

[9] Dai X., Gakidou E., Lopez A.D. (2022). Evolution of the global smoking epidemic over the past half century: strengthening the evidence base for policy action. Tob Control.

[10] Pourmemari M.H., Viikari-Juntura E., Shiri R. (2014). Smoking and carpal tunnel syndrome: a meta-analysis. Muscle Nerve.

[11] Lampainen K., Hulkkonen S., Ryhänen J., Curti S., Shiri R. (2022). Is smoking associated with carpal tunnel syndrome?. A Meta-Analysis. Healthcare.

[12] Smith G.D., Ebrahim S. (2003). Mendelian randomization': can genetic epidemiology contribute to understanding environmental determinants of disease?. Int J Epidemiol.

[13] Burgess S., Butterworth A., Thompson S.G. (2013). Mendelian randomization analysis with multiple genetic variants using summarized data. Genet Epidemiol.

[14] Hwang L.D., Lawlor D.A., Freathy R.M., Evans D.M., Warrington N.M. (2019). Using a two-sample mendelian randomization design to investigate a possible causal effect of maternal lipid concentrations on offspring birth weight. Int J Epidemiol.

[15] Liu M., Jiang Y., Wedow R., Li Y., Brazel D.M., Chen F. (2019). Association studies of up to 1.2 million individuals yield new insights into the genetic etiology of tobacco and alcohol use. Nat Genet.

[16] Hemani G., Zheng J., Elsworth B., Wade K.H., Haberland V., Baird D. (2018). The MR-Base platform supports systematic causal inference across the human phenome. Elife.

[17] Hartwig F.P., Davey Smith G., Bowden J. (2017). Robust inference in summary data mendelian randomization via the zero modal pleiotropy assumption. Int J Epidemiol.

[18] Nikolakopoulou A., Mavridis D., Salanti G. (2014). How to interpret meta-analysis models: fixed effect and random effects meta-analyses. Evid Based Ment Health.

[19] Bowden J., Davey Smith G., Burgess S. (2015). Mendelian randomization with invalid instruments: effect estimation and bias detection through Egger regression. Int J Epidemiol.

[20] Verbanck M., Chen C.Y., Neale B., Do R. (2018). Detection of widespread horizontal pleiotropy in causal relationships inferred from mendelian randomization between complex traits and diseases. Nat Genet.

[21] Hulkkonen S., Auvinen J., Miettunen J., Karppinen J., Ryhänen J. (2019). Smoking as risk factor for carpal tunnel syndrome: a birth cohort study. Muscle Nerve.

[22] Vessey M.P., Villard-Mackintosh L., Yeates D. (1990). Epidemiology of carpal tunnel syndrome in women of childbearing age. Findings in a large cohort study. Int J Epidemiol.

[23] Jung H.Y., Kong M.S., Lee S.H., Lee C.H., Oh M.K., Lee E.S. (2016). Prevalence and related characteristics of Carpal tunnel syndrome among orchardists in the Gyeongsangnam-do region. Ann Rehabil Med.

[24] Kiani J., Goharifar H., Moghimbeigi A., Azizkhani H. (2014). Prevalence and risk factors of five most common upper extremity disorders in diabetics. J Res Health Sci.

[25] Bhanderi D.J., Mishra D.G., Parikh S.M., Sharma D.B. (2017). Computer use and Carpal tunnel syndrome: a case-control study. Indian J Occup Environ Med.

[26] Fung B.K., Chan K.Y., Lam L.Y., Cheung S.Y., Choy N.K., Chu K.W. (2007). Study of wrist posture, loading and repetitive motion as risk factors for developing carpal tunnel syndrome. Hand Surg.

[27] Ulbrichtová R., Jakušová V., Osina O., Zibolenová J., Kuka S., Hudečková H. (2020). Association of the role of personal variables and nonoccupational risk factors for work-related carpal tunnel syndrome. Cent Eur J Public Health.

[28] Amankwah K.S., Kaufmann R.C., Weberg A.D. (1985). Ultrastructural changes in neonatal sciatic nerve tissue: effects of passive maternal smoking. Gynecol Obstet Invest.

[29] Winkelmann B.R., Boehm B.O., Nauck M., Kleist P., März W., Verho N.K. (2001). Cigarette smoking is independently associated with markers of endothelial dysfunction and hyperinsulinaemia in nondiabetic individuals with coronary artery disease. Curr Med Res Opin.

[30] Burke A., Fitzgerald G.A. (2003). Oxidative stress and smoking-induced vascular injury. Prog Cardiovasc Dis.

[31] Lehr H.A. (2000). Microcirculatory dysfunction induced by cigarette smoking. Microcirculation.

[32] Fuchs P.C., Nathan P.A., Myers L.D. (1991). Synovial histology in carpal tunnel syndrome. J Hand Surg Am.

