# Supplementary material for: Genetic predisposition to smoking and the risk of Carpal Tunnel Syndrome: a mendelian randomization study
Source: Clinics (Sao Paulo). 2026 Apr 11;81:100950. doi: 10.1016/j.clinsp.2026.100950 (PMC13092613; doi:10.1016/j.clinsp.2026.100950)

**CLINICS-D-25-01205_Supplementary Material**

**Supplementary Table 1** Genetic instrument details and statistical power for Mendelian randomization analyses.

**Figure S1 MR funnel plots for the associations of smoking and CTS.** (A) Funnel plots for the causal effects of cigarettes per day (past and current) and CTS. (B) Funnel plots for the causal effects of individual began smoking regularly and CTS. (C) Funnel plots for the causal effects of Smoking initiation and CTS. (D) Funnel plots for the causal effects of Current tobacco smoking and CTS. (E) Funnel plots for the causal effects of Smoking status (Never) and CTS. CTS, Carpal Tunnel Syndrome; MR, Mendelian randomization.


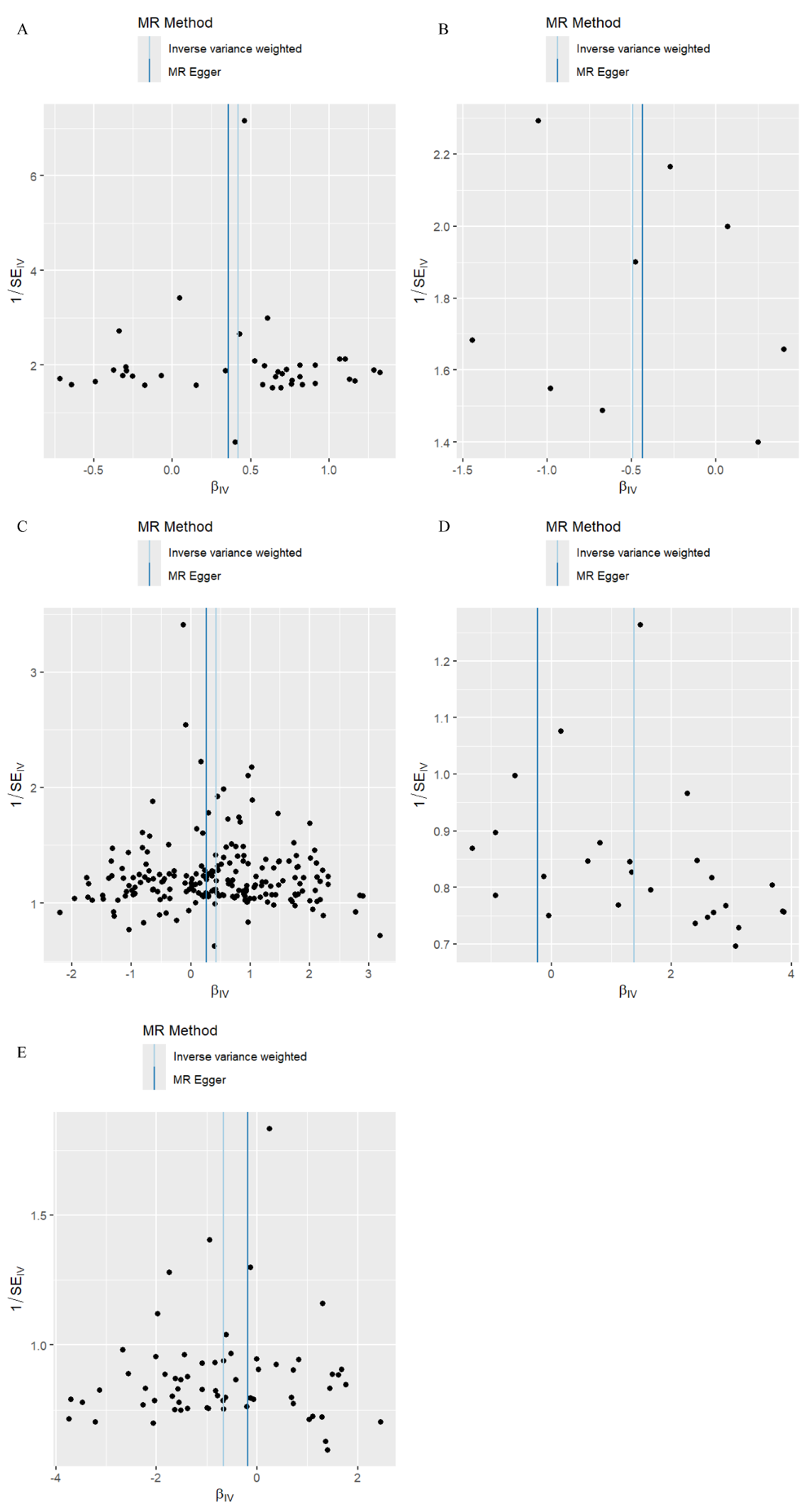


**Figure S2 MR leave-one-out plots for the associations of MDD on BPH.** (A) Leave-one-out plots for the causal effects of cigarettes per day (past and current) and CTS. (B) Leave-one-out plots for the causal effects of individual began smoking regularly and CTS. (C) Leave-one-out plots for the causal effects of Smoking initiation and CTS. (D) Leave-one-out plots for the causal effects of Current tobacco smoking and CTS. (E) Leave-one-out plots for the causal effects of Smoking status (Never) and CTS. CTS, Carpal Tunnel Syndrome; MR, Mendelian randomization.


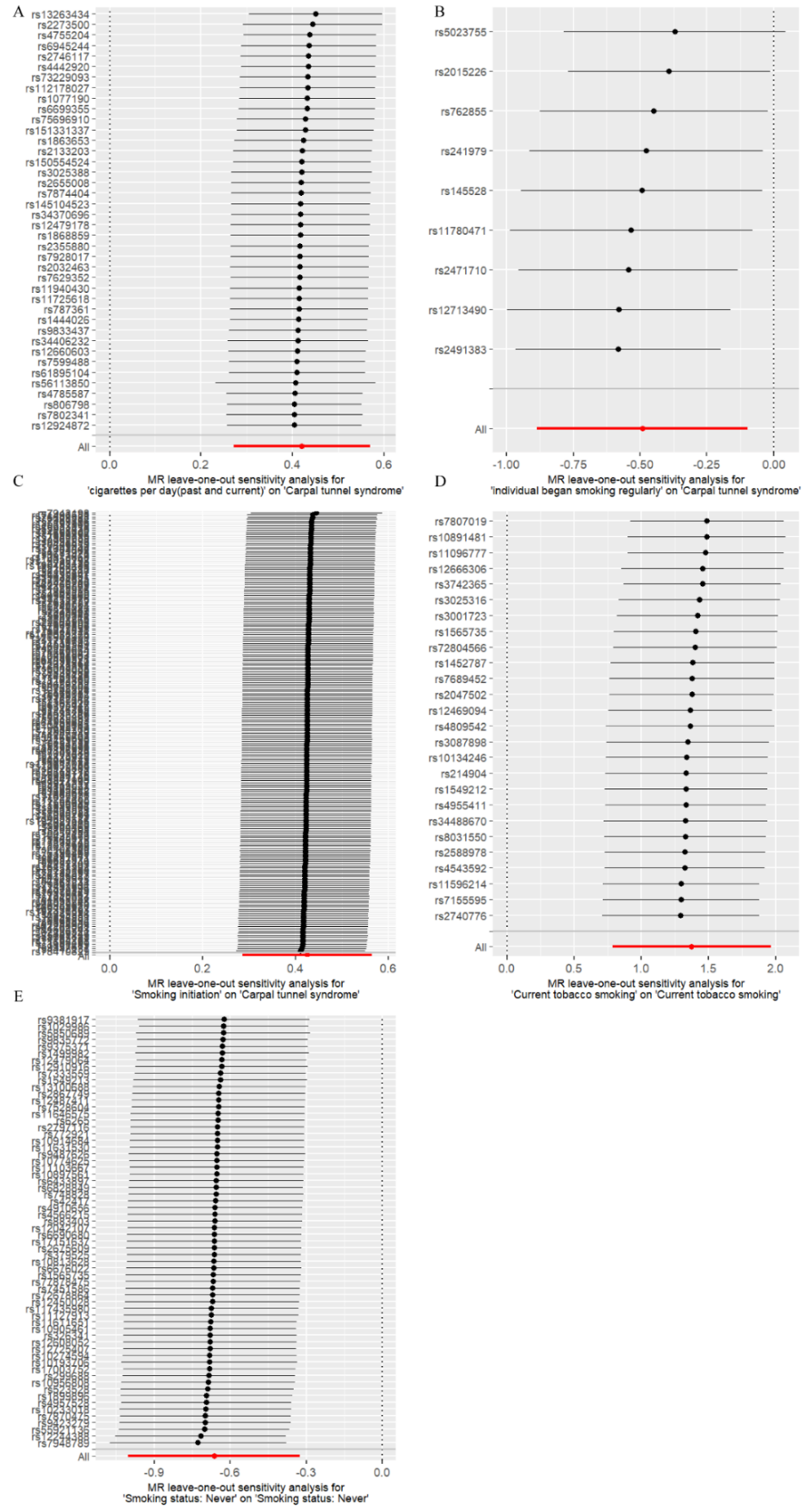

Supplement: Supplementary file 1 [file mmc1.docx]
